# Interprofessional peer-assisted learning and tutor training practices in health professions education–A snapshot of Germany

**DOI:** 10.1371/journal.pone.0278872

**Published:** 2022-12-14

**Authors:** Doreen Herinek, Robyn Woodward-Kron, Marion Huber, Stefanie M. Helmer, Mirjam Körner, Michael Ewers

**Affiliations:** 1 Charité–Universitätsmedizin, Berlin corporate member of Freie Universität Berlin and Humboldt-Universität zu Berlin, Institute of Health and Nursing Science, Berlin, Germany; 2 Department of Medical Education, University of Melbourne, Parkville, Victoria, Australia; 3 Zurich University of Applied Sciences (ZHAW), School of Health Sciences, Institute of Public Health IPH, Center for Interprofessional Learning and Practice IPLP, Winterthur, Switzerland; 4 Department 11: Human and Health Sciences, Univesity of Bremen, Bremen, Germany; 5 Medical Faculty, Albert-Ludwigs-University, Institute of Medical Psychology and Medical Sociology, Freiburg, Germany; Pondicherry Institute of Medical Sciences, INDIA

## Abstract

Peer-assisted learning (PAL)–especially peer tutorials–are gaining momentum in health professions education, particularly in interprofessional education. As little is known about the use of peer tutorials or the preparation of tutors in this context in Germany and in other countries, this study aims to provide an overview of these interventions. A cross-sectional study with a descriptive-exploratory design was conducted. German institutions for health professions education were contacted, and individuals with pedagogical responsibilities were invited to participate in an online survey. The survey was informed by two studies in which seven domains were identified as important. These included facts about the institution, the offer of PAL, the use of tutorials, and the design of tutor training. The survey used mostly closed-ended questions. The questionnaire was completed by n = 100 participants. Overall, n = 46 participants indicated that PAL was offered at their institution. Of these 46 participants, 32 (70%) indicated that uniprofessional PAL was offered, 2 (4%) indicated that interprofessional PAL was offered, and 12 (26%) indicated that both forms of PAL were offered. Peer tutoring was the most common format in both cases (73% for uniprofessional and 64% for interprofessional PAL), and mandatory interventions were mostly used to prepare the tutors. These interventions were held by educators or lecturers and were offered mostly face-to-face as workshops or as discussions. Deepening the tutors’ social competencies through training was given high relevance. Regarding content, focus was placed on communication, (self-)reflection, and group management. Eighty-eight participants expressed recommendations for future directions in terms of preparing tutors for interprofessional PAL. Minor differences between the use of uniprofessional and interprofessional PAL and tutor training were found. Nevertheless, implementation strategies varied. In future, preparatory measures for tutors should be more uniformly designed and geared to the specific requirements of uniprofessional and interprofessional learning, and that at best on an empirical basis.

## Introduction

Peer tutorials–which are a form of peer-assisted learning (PAL)–have been used in medical education around the world for many years [e.g., 1,2] and are also becoming increasingly popular in education among other health professions, such as nursing [e.g., 3]. Peer tutors aim to facilitate students’ learning and are often seen as an alternative to faculty educators [4]. Furthermore, the educational impact of peer-assisted learning stems mainly from the fact that people of nearly equal status and who are hence socially and cognitively on a similar level [5] can learn collectively without a professional educator [6]. This results–inter alia–in a deeper understanding of the material, in increased knowledge, and in skill development among tutors and tutees alike [7,8]. Peer formats are generally widely accepted and offer benefits ranging from measurable performance gains to increased self-confidence, communication, and leadership skills [4,7,8].

More recently, peer tutorials have also begun to be used to support learning “with, from and about each other” [9] among *different* health professions. In interprofessional education (IPE), peer tutorials are seen to support the development of competencies that are necessary for collaboration in clinical practice [10]. However, IPE comes with additional challenges, including the need to deal with learners’ different educational, social, and professional situations [11,12]. There is consensus among educators and researchers that IPE educators should be specifically prepared to deal with this heterogeneity [12,13]. For this reason, the literature argues that tutors who are engaged in IPE should be prepared to teach and facilitate tasks in a specific way [e.g., 14,15].

The current research situation shows that overall there is little evidence on the implementation of PAL, tutorials and tutor training, both internationally and nationally. If at all, selective initiatives can be identified, e.g., presenting their individual programs and results. Moreover, this gap is exacerbated with regard to IPE where limited evidence exists on the implementation and effectiveness of tutor training in this particular context. Regarding the international context Burgess et al. (2017) [16] investigated tutor training and learning with 115 participants from the interprofessional Peer Teacher Training (PTT) Program at the University of Sydney, Australia. The program supports participants in teaching skills as well as in comprehending other professional roles and in understanding the importance of IPE. Other research has revealed that specific tutor training has led to improved teaching skills, at least from a subjective point of view [e.g., 17]. For example, one systematic review of tutor training programs in health professions education (HPE) concluded that medicine is the dominant profession that provides tutor training and that this training is quite heterogeneous, for example, in terms of its duration and content [1,2]. Furthermore, the authors recommended that interprofessional tutor training be taken into consideration by various faculties in order to foster IPE among students of different professions [2].

Research on PAL and tutor training in HPE in Germany is also scarce. Indeed, most research in this field is limited to individual institutions and to singular professions as well. For example, one study that focused on the training of medical tutors examined the recruitment of and roles played by tutors [18]. The authors concluded that recruitment strategies and tutor roles were heterogenous but that in all cases, tutors were used to support faculty teachers [18]. Another study reported on different training strategies in skills lab tutorials, with the “How to present effectively” module being rated as the most important for the tutors’ didactic preparedness [19]. At one medical faculty in Germany, tutors received basic training for eight hours before they were allowed to conduct *interprofessional* tutorials [20]. Thematically, the training covered the topics of student moderation, presentations, group management, tutors’ roles, and the opportunity to learn how to work with simulation patients. The evaluation only covered IPE tutorials, and the tutors’ work was consistently rated positively by the higher-education students. To what extent this evaluation is related to the students’ training is unclear as the tutor training itself was not evaluated [20]. Alvarez et al. (2017) [21] studied different tutor training programs at medical faculties across Germany with a focus on the programs’ current implementation practices. The authors observed a “broad variety of training structures” [21, p. 5] and recommended that further research be conducted at additional institutions in order to gain deeper insights into this area.

Due to the lack of research, there is currently a knowledge gap concerning where, in what professions, and in what way PAL and tutor training are used. We have chosen Germany as a starting point to systematically collect and analyze data of these activities for an entire country acknowledging that these gaps also apply to international contexts. Germany could serve as an example for other countries to follow.

We investigated the use of both uniprofessional and interprofessional PAL and tutor training at several German institutions for HPE, which included institutions in the fields of medicine and in other health professions. Our aim was (1) to gain deeper insights into the use of PAL and the practices of tutor training at these institutions in order to provide an initial snapshot of Germany but at the same time to stimulate international debate on the topic and (2) to lay the foundation for an in-depth exploration of the didactic design, implementation, and anticipated effects of (especially interprofessional) peer tutor training.

Our research questions were how peer-assisted learning is used in general–and in interprofessional education in particular–in pre-registration health professions education in Germany and how tutors are prepared there for uniprofessional and interprofessional tutorials. Next to this, we were interested in recommendations for adequate tutor training if no separate preparation for interprofessional tutorials is currently offered.

## Material and methods

### Context and design

A cross-sectional online survey was carried out using a descriptive-exploratory design as the first part of the multi-part Prep4TUT-project (Preparation For a Tutoring Activity for Interprofessional Peer-Assisted Learning). The methods and results of this first part of the project are presented here. The second part of the project is designed to elicit tutors’ perspectives (i.e., via group discussions with tutors), while the third part will capture international expert perspectives (i.e., via expert interviews) [22]. Both the second and third studies are currently ongoing, and their procedures and results will be published at a later date.

The Prep4TUT-project as a whole was approved by the independent Medical Ethics Committee of Charité–Universitätsmedizin Berlin (EA1/270/20). All participants (only adults) in this study were asked to provide written consent via the first page of our questionnaire where we informed potential participants about data protection policies, voluntary participation, and analyses based upon an anonymous dataset. Participants had to select a “yes”-button to provide consent in order to proceed.

### Instrument

An online questionnaire in German was used to gather data in this study (see S1 File). The questionnaire was developed based on two studies; one empirical study [18] and one systematic review [2]. It was tested in an extended research team in terms of its structure, comprehensibility, and manageability, and it was subsequently revised. The questionnaire was estimated to require about 15 minutes to complete and covered the following seven domains. The first domain covered the institutional context and information about the respondent as a representative of the institution. The second domain was about the general provision of PAL at the institution. In the third domain participants were asked for the provision of uniprofessional PAL and in the fourth for the provision of interprofessional PAL at their institution. The fifth and sixth domain contained questions on the preparation of students as tutors for uniprofessional or interprofessional tutorials. And the last domain included the recommended preparation of students as tutors for interprofessional tutorials if no separate training was offered.

The questionnaire used different types of questions and various response formats, which we will briefly describe here (for more detail see S1 File). The first domain contained questions about some basic information on the respondent as a representative of the institution such as duration of employment at this institution, current function (multiple answer option to choose between research, teaching, administration, others), and whether the respondent is responsible for PAL (single answer option to choose between yes or no). In this domain questions about institutional contexts like type of institution (single answer option: university, university of applied sciences, vocational school, others), sponsorship of the institution (single answer option: private, public, independent–non-profit, independent–private commercial), which professions are trained at this institution (multiple answer option) and about the cohort sizes studying there (single answer option: 1–99, 100–499, 500–999, >1000, not specified) were asked. In the second domain we asked if PAL is offered generally (filter question, single answer option to choose between yes and no) and if so in which manner (single answer option to choose between uniprofessional, interprofessional or both, uni- and interprofessional). The third and fourth domains thus came into play as soon as it was specified that PAL was offered. Here, questions were then specifically asked about uniprofessional, interprofessional offers, or both. In this domain three topics were included: (1.) the professions in which PAL is offered and at what time (multi-answer option), (2.) fields of application (multi-answer option) and (3.) applied formats (filter question, multi-answer option to choose between peer tutorials, peer assessment and peer mentoring). As soon as tutorials were selected as applied format, the questions of domain 5 opened for uniprofessional tutorials and 6 for interprofessional tutorials, respectively, which were to obtain information about the offered tutor’ preparation. All respondents from institutions that do not offer interprofessional tutorials were asked to answer questions about recommendations for future directions regarding tutor preparation for interprofessional tutorials (Domain 7). Hence domain 5, 6 and 7 contained the same questions but with different foci. The first question was if preparation for tutors is/should be offered (filter question, single answer option to choose between yes and no). Afterwards, the offered preparation was specifically addressed, e.g. by asking whether the preparation is/should be voluntary or mandatory (single answer option), in which department the measures are/should be located (single answer option), who (should) carry them out (multi-answer-option), how long they (should) last (free text), which contents are/should be addressed (multi-answer-option) and in which format they are/should be offered (multi-answer-option). Finally, the last question was which competencies should be acquired on the part of the tutors and thus are considered most important for future tutoring (6-point likert scale ranging from 1: very unimportant to 6: very important).

### Data collection

Data collection took place in Germany at institutions for preregistration education in medicine, nursing, obstetrics, physiotherapy, occupational therapy, and speech & language therapy, which represent the largest occupational groups in the German healthcare system. Moreover, these professions are most often discussed in terms of potential professionalization and are usually included in IPE in Germany. Of these professions, only physicians and obstetricians are regularly educated at the graduate level, whereas most nurses, physiotherapists, occupational therapists, and speech & language therapists become qualified through vocational training. Therefore, in addition to tertiary-level institutions (universities and universities of applied science), secondary-level institutions (vocational schools for health professions education) also had to be considered for data collection.

In Germany there is no complete list of all these educational institutions that offer preregistration education in the above mentioned professions. In order to create a list of educational institutions that offer preregistration education in the above-mentioned professions, websites of professional and specialist associations as well as ministries of the federal states in Germany were screened between November 2019 and June 2020. The extensive search resulted in 1,196 entries after removing duplicates. All eligible institutions were contacted via email and asked to forward the invitation to participate (along with a link to the online survey) to the respective individual(s) responsible for PAL activities. Participation in the online survey was voluntary. The online survey was accessible for three weeks. Subsequently, an electronic reminder was sent, after which the survey remained accessible for another week. Data collection was performed during the first year of the COVID-19 pandemic, but the findings are assumed to be applicable to circumstances prior to the pandemic.

### Data processing and analysis

Since all analyses are considered exploratory and there was no goal of achieving representative data, no sample size was calculated. A total of n = 180 participants answered the questionnaire, 80 of whom gave incomplete answers. Most of these respondents dropped out at early stages of the questionnaire (50 did not answer more than five questions). Since most of the variables used in the questionnaire are important for this study, we decided to include only complete datasets in the analysis (n = 100). A response rate cannot be calculated because we do not know (1) how many people were actually reached by email or (2) how many of the recipients forwarded the invitation to how many fellow colleagues.

The data (see S2 File) were processed and analyzed with IBM SPSS v27.0 using descriptive statistics. The individual parameters were calculated using frequencies, relative frequencies, and mean values. The figures were created with Microsoft Excel (Office 365).

## Results

**Fig 1:**
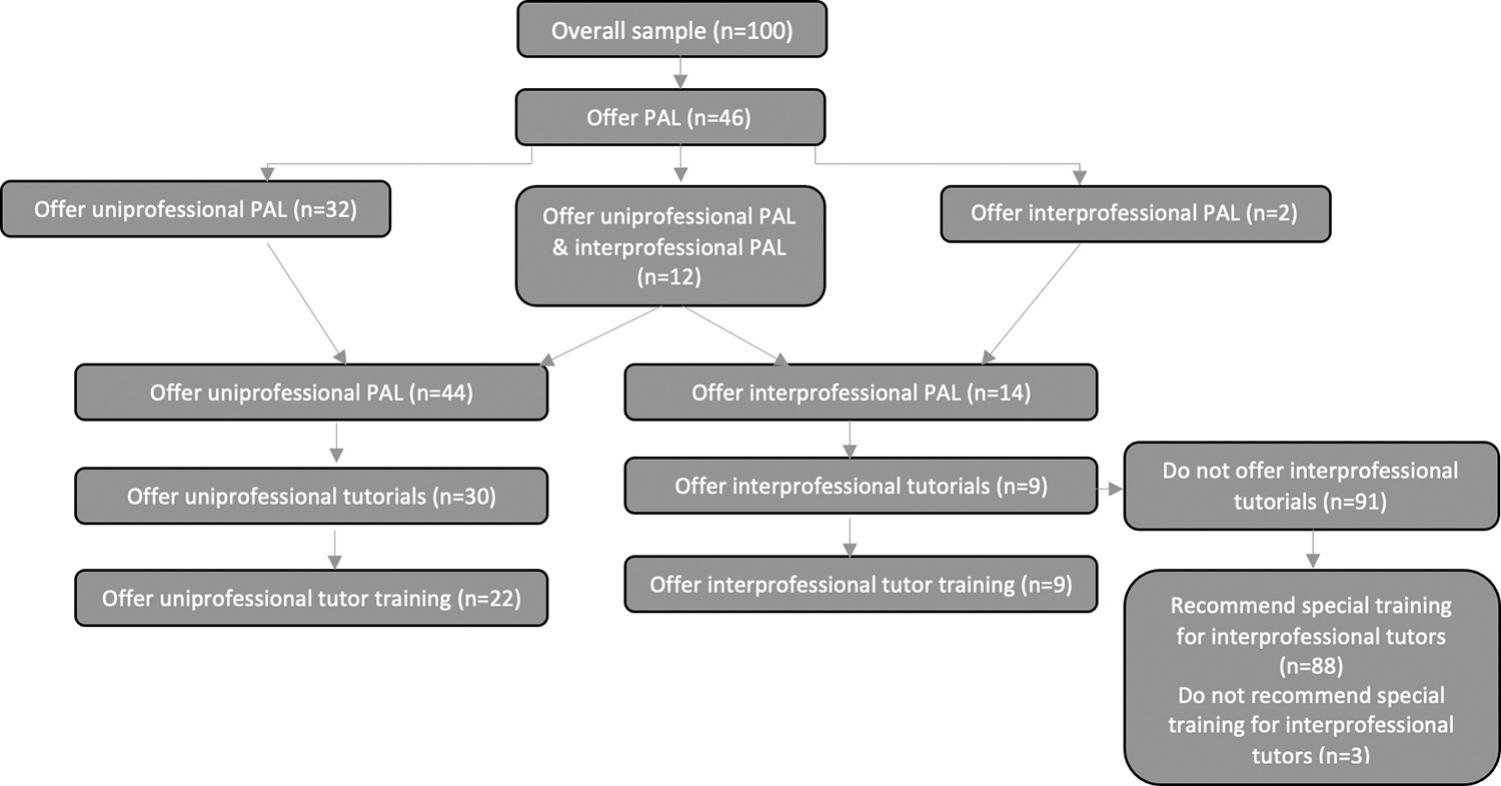
Overview of sub-samples.

Results of the online survey are presented based on the following sub-samples (see Fig 1).

### Sample

Of the 100 participating respondents, 99 provided information on their type of institution and sponsorship. Two-thirds (n = 74) of respondents came from the field of vocational education (i.e. secondary education); 20 participants came from the tertiary education sector, which includes universities (n = 11) and universities of applied sciences (n = 9). In the case of the tertiary sector, all institutions are publicly funded whereas vocational schools in the secondary education level are mostly independent and non-profit sponsored (n = 41), and some are public funded (n = 15).

As can be seen in Table 1, a wide variety of occupational groups are trained at the surveyed institutions. Nursing and physiotherapy were particularly well represented, although the number of nursing students / trainees in the institutions was rather low (i.e., fewer than 500 students). In the other therapy professions and in obstetrics, the number of learners was mostly limited to 99 or fewer. In the academically trained professions, "unknown" was often given as a response. Mainly large institutions (>1,000 learners) were recorded for medicine.

**Table 1:** Professions trained at institutions by number of learners in total [multiple answer options, sample n = 100].

Profession	Number of learners in total					
	1–99	100–499	500–999	> 1,000	unknown	total
Physiotherapy	17	7	-	-	2	**26**
Dentistry	1	2	-	-	2	**5**
Pharmacy	-	1	-	-	-	**1**
Psychology / psychotherapy	1	-	-	-	9	**10**
Nursing	30	46	1	1	1	**79**
Medicine	1	1	-	8	8	**18**
Occupational therapy	11	1	-	-	2	**14**
Speech therapy / clin. linguistics	1	-	-	-	9	**10**
Obstetrics	12	-	1	-	-	**13**
Other	9	4	1	1	-	**15**

Most respondents (n = 94) reported that they were involved in teaching as their main role, and a few indicated that their primarily role was research (n = 9), administration (n = 18), or other (n = 15). The role of “other” included being a leader, student, clinician, or preceptor. Twenty-two respondents were responsible for PAL at their institution or within their department. The average length of employment at a respondent’s current institution was 13.4 years (ranging from <1 to 43 years; median: 17).

### The general practice of PAL

Nearly half of all respondents (n = 46) indicated that their institution offered PAL in some way. Of these 46 respondents, n = 32 (70%) indicated that their institution provided uniprofessional PAL, n = 2 (4%) indicated that their institution provided interprofessional PAL, and n = 12 (26%) indicated that their institution provided both forms of PAL. Uniprofessional PAL was mostly offered by vocational schools (n = 28), while interprofessional PAL was mainly offered at universities or universities of applied sciences (n = 8).

### Uniprofessional PAL: The practice

Of the 100 total respondents, 44 reported that uniprofessional PAL was offered at their institution (32 of whom reported that only uniprofessional PAL was offered and 12 of whom reported that both forms of PAL were offered). These 44 respondents are used as a sub-sample in the following analyses (see also Fig 1).

The 44 participants were asked which professions the uniprofessional PAL was offered in at their institutions. As the institutions often offered training in more professions than the ones that were mainly included in the study, additional professions were explicitly asked about. According to n = 42 (95%) of participants, uniprofessional PAL was offered predominantly in all or several semesters in each surveyed profession, except for pharmacy (see Table 2).

**Table 2:** Uniprofessional PAL offers per profession and timing [multiple answer options, sample n = 40].

Profession	Timing		
	in one semester	in several semesters	in all semesters
Physiotherapy n = 3	-	-	3
Dentistry n = 1	-	-	1
Pharmacy	-	-	-
Psychology / psychotherapy n = 1	-	1	-
Nursing n = 30	3	10	17
Medicine n = 6	-	3	3
Occupational therapy n = 1	1	-	-
Speech therapy / clin. linguistics n = 1	1	-	-
Obstetrics n = 2	-	1	1
Other n = 3	-	1	2

**Fig 2:**
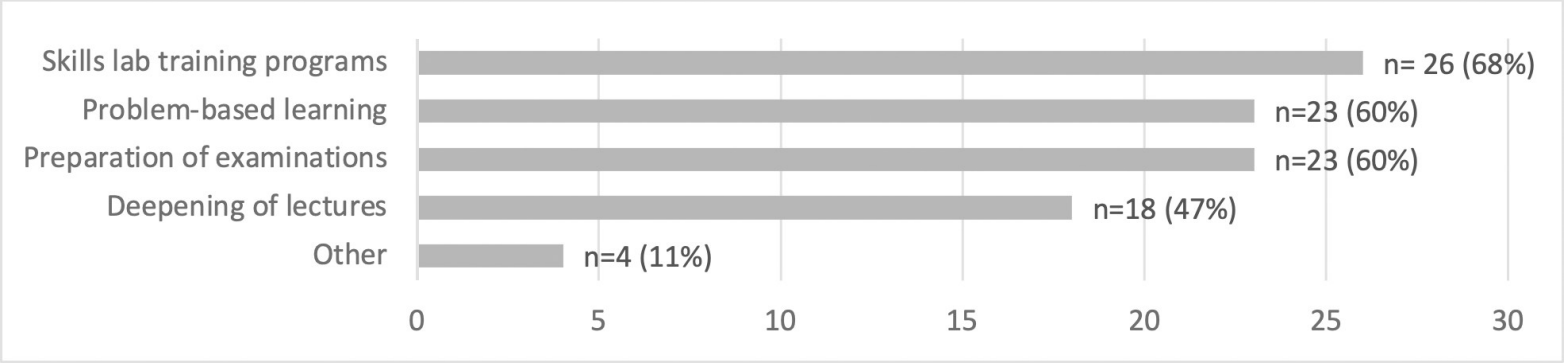
Fields of application of uniprofessional PAL at the institution [multiple answer options, sample n = 38].

Uniprofessional PAL was reportedly used in a range of didactic settings / learning environments by n = 38 respondents, especially in the context of skills labs as well as in problem-based learning and in preparation for exams. Uniprofessional PAL was also commonly used to deepen the content of lectures (see Fig 2). A project entitled “Learners Instruct Learners” as well as orientation weeks for students / trainees at the beginning of their studies were listed among “other.”

Of all 44 participants, n = 41 (93%) indicated that uniprofessional PAL was used in a special format. We specifically asked for the formats peer tutorials, peer mentoring and peer assessment. In total peer tutorials were being most frequently mentioned (n = 30; 73%), followed by peer mentoring (n = 14; 34%) and peer assessment (n = 14; 34%).

### Uniprofessional PAL: Tutor preparation

Of the n = 30 respondents who indicated that their institution offered tutorials, n = 22 (73%) indicated that their institution also provided some kind of intervention (e.g., tutor training courses or other preparatory measures) for its tutors. Of the participants from institutions that offered such intervention, n = 9 (41%) stated the intervention was voluntary, and n = 13 (59%) stated it was mandatory. On average, the duration of the intervention was 24 hours; however, a considerable range of between 1 and 200 hours was found (median: 8).

Half of all respondents that indicated that their institution provided some kind of intervention (n = 11) mentioned that teachers, lecturers, and managers were responsible for the intervention instead of departments (e.g., learning centers, continuing education, or the associate dean of studies / the department of study). Respondents indicated that the training was carried out by variously qualified individuals, who were mostly pedagogues (n = 12) and lecturers (n = 11) as well as clinicians (n = 6). More experienced tutors were also stated to conduct the training programs.

According to n = 21 respondents, most of the training programs were conducted as face-to-face meetings between tutors and the person responsible for the uniprofessional PAL at the institution (n = 15), followed by skills training (n = 12). Lectures were mentioned as being most often offered as a combination of face-to-face and online teaching (n = 3).

**Fig 3:**
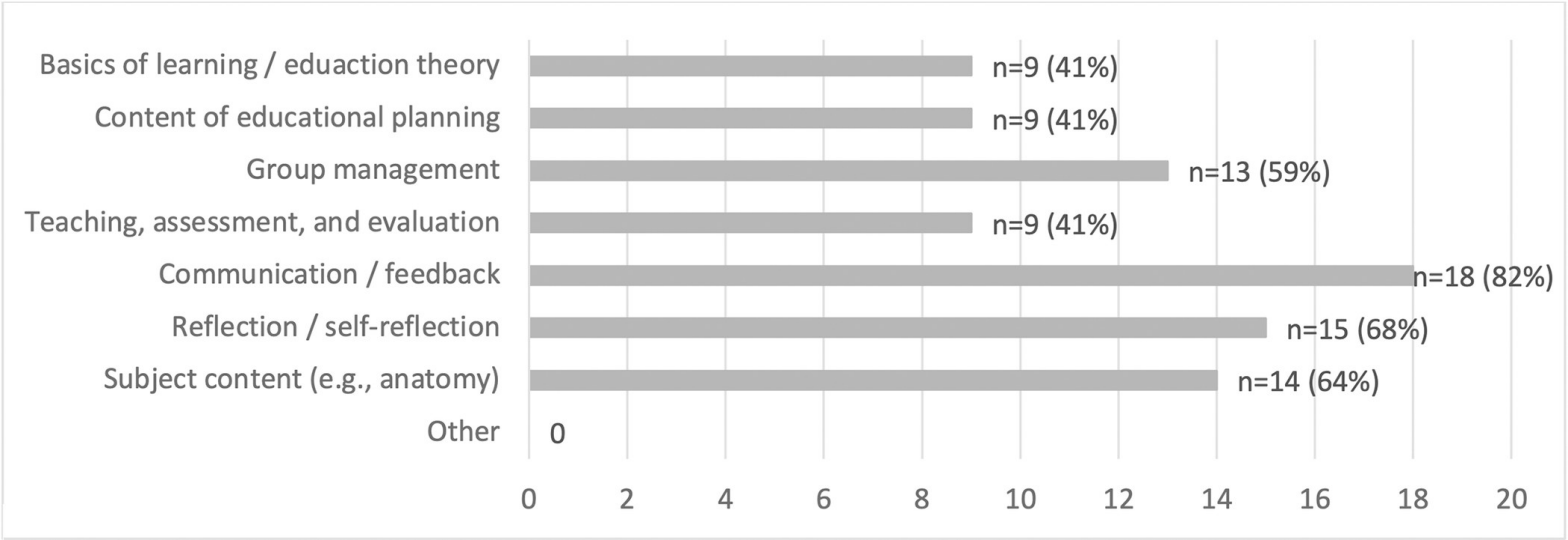
Content of intervention [multiple answer options, sample n = 22].

The content mainly involved communication / feedback, (self-)reflection, subject content, and group management (see Fig 3).

Respondents were also asked which of the competencies that were acquired through the training were most relevant. Of n = 22, social competence was rated highest (mean: 5.2 out of 6), followed by teaching competence (mean: 4.9 out of 6), personal competence (mean: 4.8 out of 6), and professional competence (mean: 4.7 out of 6).

### Interprofessional PAL: The practice

Only a small portion of the total of 100 valid records referred to interprofessional PAL. Fourteen individuals stated that they ran this form of interprofessional tutorial at their institution, whereas two offered interprofessional PAL exclusively, the remaining 12 offered both interprofessional PAL and uniprofessional PAL. In total, interprofessional PAL was offered among most of the professions, except dentistry and psychology (see Table 3).

**Table 3:** Interprofessional PAL offers per profession and timing [multiple answer options, sample n = 11].

Profession	Timing		
	in one semester	in several semesters	in all semesters
Physiotherapy n = 3	3	-	-
Dentistry	-	-	-
Pharmacy n = 1	1	-	-
Psychology / psychotherapy	-	-	-
Nursing n = 7	3	2	2
Medicine n = 3	-	3	-
Occupational therapy n = 2	2	-	-
Speech therapy / clin. linguistics n = 2	2	-	-
Obstetrics n = 1	-	1	-
Other	-	-	-

**Fig 4:**
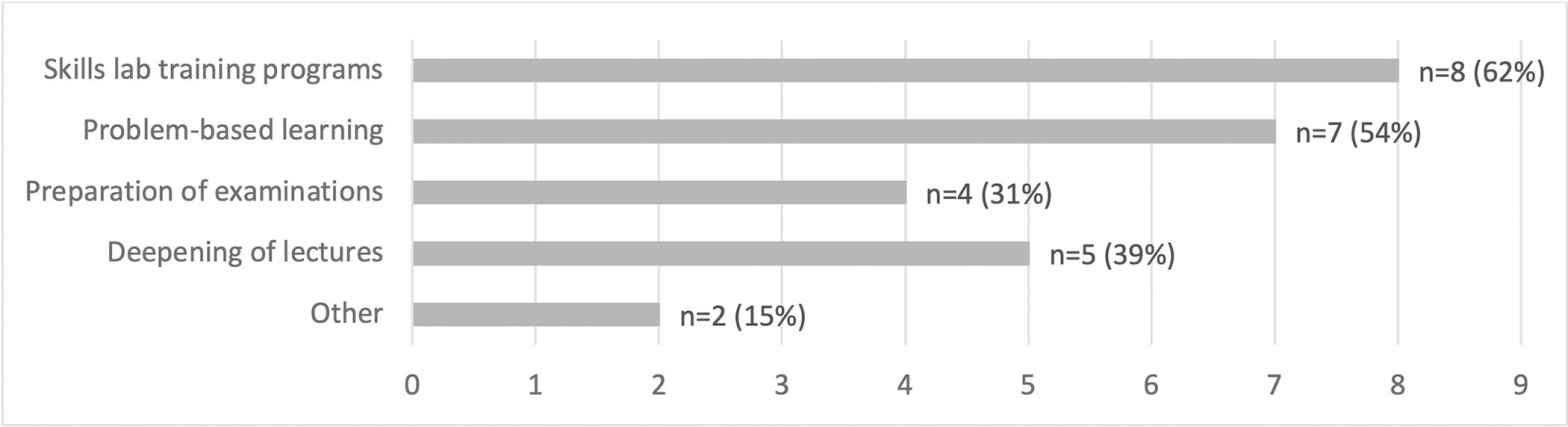
Fields of application of interprofessional PAL at institutions [multiple answer options, sample n = 12].

Interprofessional PAL was used in almost all mentioned didactic settings / learning environments, and mostly in skills lab training programs (see Fig 4).

The question about the applied formats of interprofessional PAL was answered by a total of n = 14. Most of the participants in this subgroup indicated that interprofessional PAL is offered in the form of peer tutorials (n = 9). Three participants applied interprofessional PAL in the form of peer assessment and n = 2 in the form of peer mentoring.

### Interprofessional PAL: Tutor preparation

Of the nine respondents who self-reported that their institution offered peer tutorials, n = 7 stated that tutor preparation was compulsory whereas n = 2 stated that tutor preparation was voluntary. In most cases (n = 4), the associate dean of studies / the department of study was indicated as being responsible for organizing the tutor training programs, but the current implementation was the responsibility of the educators (n = 7), the lecturers (n = 4), and/or the clinicians (n = 4). The tutor training programs mostly took place face-to-face as a workshop (n = 8), followed by face-to-face discussions between tutors and the person responsible for the PAL on the part of the institution (n = 6). Skills training and lectures were only held face-to-face while online lectures and a combination of face-to-face and online lectures were very rarely selected overall.

The average duration of the interventions was 16 hours (ranging between 6 and 60 hours; median: 8).

**Fig 5:**
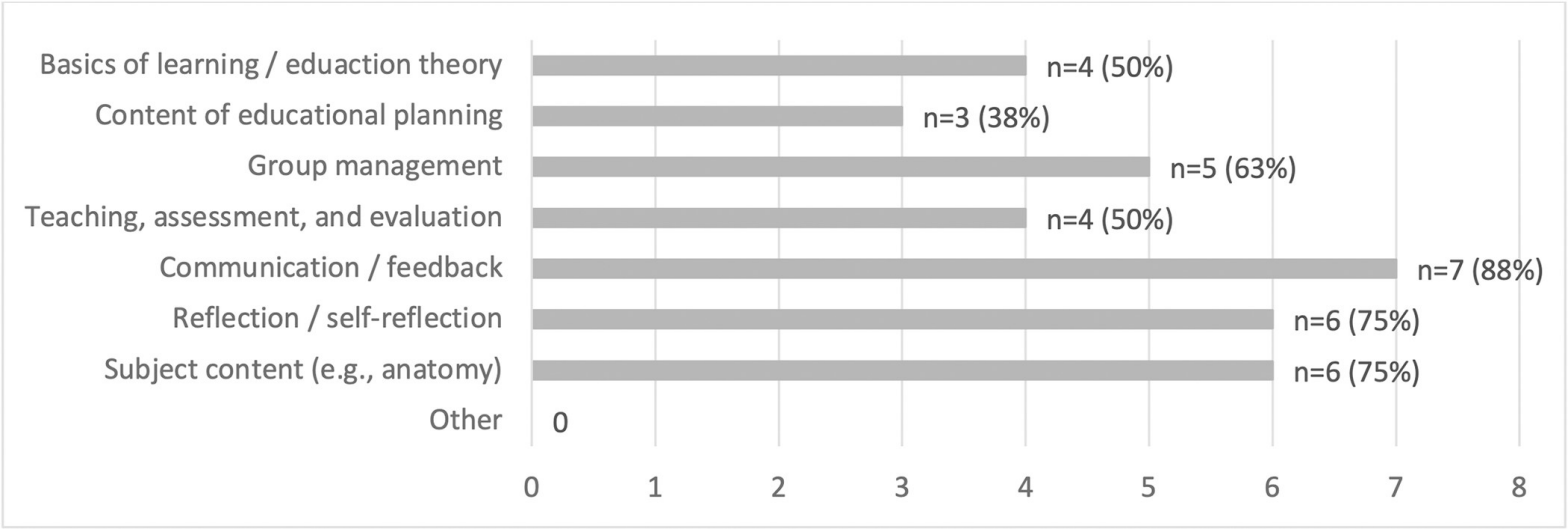
Content of preparatory measures [multiple answer options, sample n = 7].

Tutor training content also overlapped and was not merely offered in isolation; instead, it was combined with communication / feedback, (self-)reflection, subject content, and group management as the main reported content (see Fig 5).

Regarding the tutors’ competencies that were considered relevant for conducting tutorials successfully in an interprofessional context, teaching competence was given the highest relevance (n = 8; mean: 4.8 out of 6) and was closely followed by social competence (n = 8; mean: 4.6 out of 6). Personal competence was also rated as important (n = 8; mean: 4.3 out of 6), and professional competence was given the lowest relevance (n = 7; mean: 3.7 out of 6).

### Interprofessional PAL: Future directions for tutor training

Most respondents made recommendations for tutor training for IPE as there aren´t any IPE tutorials and tutor training offers so far at their institutions. These 91 respondents were asked to indicate their views on the future design of tutor training for IPE tutors.

The question of whether tutors should be specially prepared for interprofessional PAL was affirmed by n = 88 (97%) of respondents, whereas only n = 3 (3%) disagreed. Of those who considered preparation useful, n = 53 advocated for a mandatory preparation course, while n = 35 reported that a voluntary offer would be sufficient. The average ideal duration of training was estimated by n = 80 of participants to be about 53 hours, with the lowest value being two hours and the highest value being 800 hours.

Of the n = 88 respondents who gave feedback on the question as to which department should be responsible for organizing tutor training, n = 34 recommended that learning centers be made responsible, whereas only n = 24 respondents recommended that institutions for continuing education be made responsible, n = 18 recommended that the associate dean of studies / the department of study be made responsible, and n = 12 recommended that other departments be made responsible. Many participants indicated that they would prefer–by a wide margin compared with the other options–that the PAL should be conducted by qualified pedagogues (n = 64) or lecturers (n = 52).

**Fig 6:**
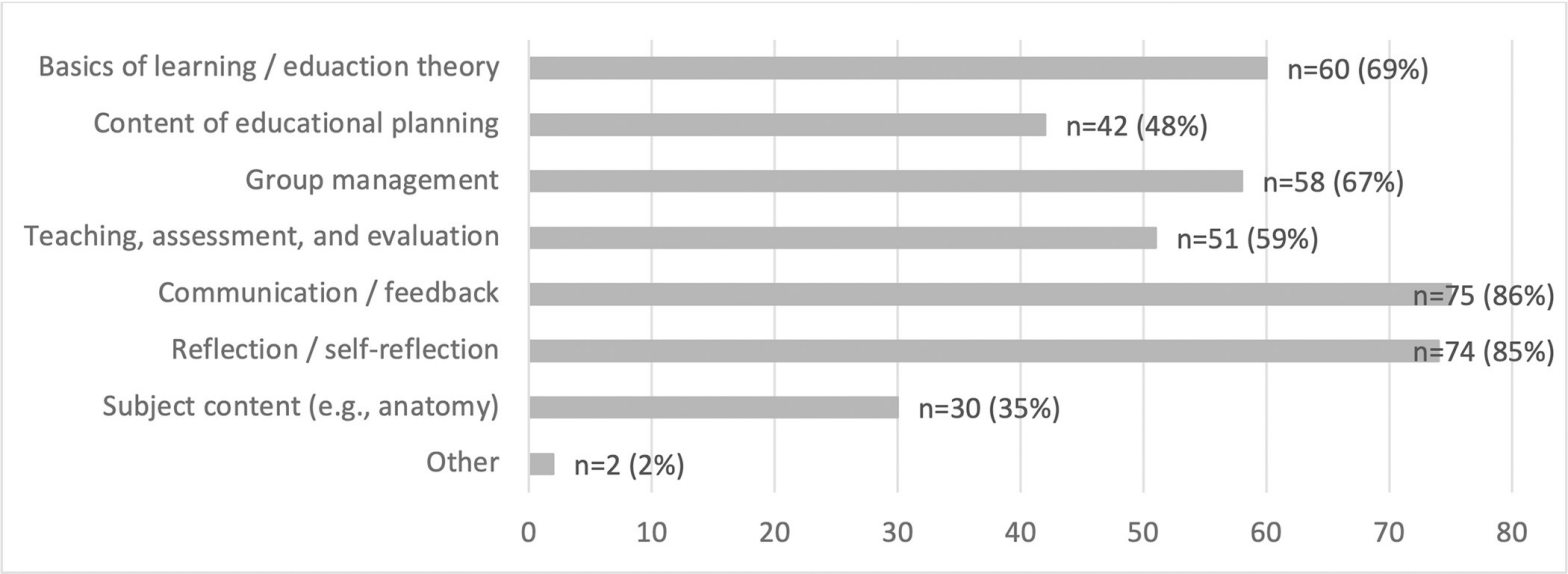
Content of preparatory measures [multiple answer options, sample n = 86].

It was argued that the training programs should take place face-to-face. A combination of face-to-face and online elements was also deemed acceptable, if necessary. Participants recommended that the training programs should be provided as workshops (n = 80) or meetings between tutors and the person responsible for the PAL on the part of the institution (n = 69). They also recommended focusing on communication / feedback as well as on (self-)reflection in terms of content. Basics of learning / education theories and group management were also considered important (see Fig 6).

In terms of relevant competencies, n = 86 respondents rated social competence the most important (mean: 5 out of 6), followed by teaching and personal competence (both: mean: 4.9 out of 6). Professional competence was rated the least important, although still important (mean: 4.5 out of 6).

## Discussion

This study aimed to provide first insights into the use of PAL and tutor training practices which can be a starting point for an in-depth discussion on common practices and on didactic design of tutor training and PAL activities. The snapshot findings from Germany suggest that PAL is used in secondary and tertiary preregistration education in various health professions and not predominantly in medicine–a finding that stands in contrast to what the majority of the literature suggests [1,2]. In this context, PAL is mostly offered in the form of tutorials and is used to stimulate both uniprofessional and interprofessional learning. This finding is in line with the trend of increasingly often using learner-centered approaches in HPE [23,24] and may also be related to the anticipated positive effects of PAL on tutors’ and tutees’ learning, including improved cognitive, psychomotor, and affective development [7].

Almost half of all respondents stated that PAL was currently being offered at their institution in various professions. However, it is not known whether these offers had only emerged recently. Nevertheless, Germany seems to be slowly catching up to with other countries in which PAL has long been used successfully in various training programs [7,10], thereby also leading to the increased use of interprofessional PAL.

Based on the data collected in this study, uniprofessional PAL is the most commonly chosen format in the vocational training of nurses, obstetricians, and allied health professions, and it is usually offered over several semesters. In the higher-education context, on the other hand, interprofessional PAL is used more frequently but is usually limited to one semester. This finding can be explained by the fact that the vocational schools for these professions are often smaller institutions that train only one profession. In Germany, the international discourse on IPE is mainly taken up at universities and places special focus on physicians and on their traditionally leading role in healthcare [25], which could explain why offers to prepare learners for collaborative practices such as interprofessional PAL began to be taken up earlier at these universities. The well-documented challenges of IPE–including inflexible curricula and cohorts of different sizes in the individual professions [26,27]–might also have influenced this result.

Most of the respondents found mandatory preparation in the form of structured offers to be important. Accordingly, the majority of tutors were prepared for their role as tutors. This is common practice in medicine and may therefore have been widely adopted. However, in line with previous research findings [1,21], the preparation of tutors displays a wide range of variation. Calls for more standardized approaches for preparation have thus far gone unheeded.

It is particularly noteworthy that preparations for uniprofessional PAL and interprofessional PAL tutors do not differ significantly, although a clear majority of respondents also recommend that tutors should undergo a specific / different preparation for interprofessional than for uniprofessional tutorials. These recommendations are in line with initial assumptions known from the German literature about tutor training programs for interprofessional PAL [14,15] but also from international contexts like the Peer Teacher Training Program, at University of Sydney [16]. However, it remains unclear why these recommendations have not been implemented more widely. The opportunity to specifically preparing tutors–and subsequently also students–for interprofessional teamwork and collaborative practice has so far remained unexplored or limited to isolated activities. This snapshot of Germany should help to give visibility to some of these activities. Furthermore, the results of this study should initiate a debate on how PAL can best be implemented and how tutors can be profitably trained in different contexts thus contributing to the development of good practice.

### Strengths and limitations

This study provided insights into a hitherto under-researched field of health professions education in Germany. The study expands upon existing knowledge about the implementation of PAL and the practice of tutor training in this context. However, the results of this study could not be representative of all institutions of health professions education in Germany. Instead, it was necessary to conduct a broad-based recruitment and casual sample which cannot claim to be representative because there is no defined list of accredited preregistration programs from which such a sample could have been drawn. It was also unclear in advance who in the individual organizations was responsible or able to provide information on PAL or on tutor training issues. Therefore, our data were collected via a voluntary online survey.

Out of a total of 1,196 institutions in Germany that were contacted, only 100 fully completed questionnaires were received, which meant that the response to this online survey was low. The rate could not be increased even despite considerable efforts such as sending multiple reminders, asking for forwarding to other persons responsible as well as extending the survey period. It was also not possible to rule out the notion that several people from one department at the same institution took part in the survey. The fact that the data collection took place in the midst of the Covid-19 pandemic may also have had an influence on the response rate and thus at least partly explain the low response rate. Furthermore, there was no possibility to make the survey binding or to directly address the respective responsible persons in the facilities. It is also possible that the structures at institutions are not clear and that the contacted individuals were thus not aware of where to send the questionnaire within the respective institutions.

Due to its reliance on self-reported data, our study might be subject to bias. To mitigate this bias, a confidential online survey was used.

As interprofessional PAL is not yet widespread in Germany, we asked participants for their recommendations for IPE tutor training, which represents a more theoretical level of investigation. Nevertheless, these results provided us with foundational findings and recommendations that can be used to guide the other two parts of the Prep4TUT-project mentioned above as well as other research on this topic.

## Conclusion and outlook

In the future, increased efforts will need to be made in order to further investigate the empirical practice of PAL in health professions education. Although Germany serves as an example here, the topics PAL and tutor training are also highly relevant internationally. It is worth to take a closer look on how educational institutions in other countries use PAL and how they prepare their tutors for their tasks. This overview could serve to initiate exchange on this topic, identify good practice and learn more about the effects of tutor training practices and the impact of PAL. On this basis, it might be of interest to find some kind of “consensus” how tutors should be prepared for either uniprofessional or interprofessional PAL via a deeper educational reflection.

It remains unclear if the current heterogeneity in the use of PAL and in the preparation of tutors is a coincidental finding or if it is conceptually justified. Either more standardization of PAL and tutor training would have to be considered or the conceptual ideas of PAL would have to be made explicit. Also, the question of how tutors can be specifically prepared to promote interprofessional collaboration needs to be analyzed in greater detail. In a multi perspective approach qualitative surveys will be conducted. For example, interprofessional PAL tutors will be asked in focus groups about their experience with and need for training. Expert interviews will also help to uncover how interprofessional PAL or tutor preparation could be tailored to help meet the goals of IPE and of collaborative practice in this particular context. Such tailoring might help to make the use of PAL in general and interprofessional PAL in particular in HPE–as well as the training of tutors who work in this context–more theory-led and evidence-based and thus also more effective in the future.

## Supporting information

S1 File German and English language questionnaire. (TIFF)

S2 File Dataset anonymous. (SAV)

## References

[1] Burgess A, McGregor D, Mellis C. Medical students as peer tutors: a systematic review. BMC Medical Educ. 2014 Jun 14: 115. doi: 10.1186/1472-6920-14-115 24912500 PMC4237985

[2] Burgess A, McGregor D. Peer teacher training for health professional students: a systematic review of formal programs. BMC Medical Educ. 2018; 18: 263. doi: 10.1186/s12909-018-1356-2 30442139 PMC6238310

[3] Carey MC, Kent B, Latour JM. Experiences of undergraduate nursing students in peer assisted learning in clinical practice: a qualitative systematic review. JBI database of systematic reviews and implementation reports. 2018; 16 (5): 1190–1219. doi: 10.11124/JBISRIR-2016-003295 29762313

[4] Schuetz E, Obirei B, Salat D, Scholz J, Hann D, Dethleffsen K. A large-scale peer teaching programme–acceptance and benefit. ZEFQ. 2017; 125: 71–79. doi: 10.1016/j.zefq.2017.05.026 28599822

[5] Lockspeiser TM, O’Sullivan P, Teherani A, Muller J. Understanding the experience of being taught by peers: the value of social and cognitive congruence. Health Sci Educ Theory Pract. 2008; 13(3): 361–72. doi: 10.1007/s10459-006-9049-8 17124627

[6] Topping KJ, Ehly SW. Introduction to Peer-Assisted Learning. In: Topping KJ, Ehly SW: Peer-assisted learning. Mahwah, NJ: L. Erlbaum Associates; 1998. pp. 1–23.

[7] Secomb J. A systematic review of peer teaching and learning in clinical education. J. Clin. Nurs. 2008; 17: 703–716. doi: 10.1111/j.1365-2702.2007.01954.x 18047577

[8] Williams B, Reddy P. Does peer-assisted learning improve academic performance? A scoping review. Nurse Educ. Today. 2016; 42: 23–29. doi: 10.1016/j.nedt.2016.03.024 27237348

[9] CAIPE–Centre for the Advancement of Interprofessional Education. Interprofessional Education: Today, Yesterday and Tomorrow. Fareham UK: CAIPE. 2002. Avalaible from: https://www.caipe.org/download/caipe-2002-interprofessional-education-today-yester-day-and-tomorrow-barr-h-pdf/.

[10] Sevenhuysen S, Thorpe J, Molloy E, Keating J, Haines T. Peer-Assisted Learning in Education of Allied Health Professional Students in the Clinical Setting: A Systematic Review. J Allied Health. 2017; Spring 46(1): 26–35. . 28255594

[11] El-Awaisi A, Hajj SE, Joseph M, Diack L. Perspectives of pharmacy students in Qatar toward interprofessional education and collaborative practice: A mixed methods study. J. Interprof. Care. 2018; 32(6): 674–688. doi: 10.1080/13561820.2018.1498466 30052106

[12] Howkins E, Bray J. Preparing for Interprofessional Teaching. Oxon: Radcliffe Publishing Ltd; 2008.

[13] Botma Y. Consensus on interprofessional facilitator capabilities. J. Interprof. Care. 2019; 33 (3): 277–279. doi: 10.1080/13561820.2018.1544546 30422009

[14] Hundertmark J, Homberg A, Alvarez S, Lauber H, Berger S, Büscher C, et al. Practice Report / Bericht aus der Praxis: Tutor training for a peer-assisted interprofessional communication seminar: A work in progress. ZEFQ. 2017 May 122: 61–63. doi: 10.1016/j.zefq.2017.04.003 28478890

[15] Ringel N, Maatouk Bürmann B, Fellmer-Drueg E, Roos M, Herzog W, Nikendei C, et al. Integriertes Peer Teaching klinischer und kommunikativer Kompetenzen. Wie bereiten wir studentische Tutoren darauf vor? Psychother Psychosom Med Psychol. 2015; 65(8): 288–295. doi: 10.1055/s-0034-1398549 25794354

[16] Burgess A, Roberts C, van Diggele C, Craig M. Peer teacher training (PTT) program for health professional students: interprofessional and flipped learning. BMC Medical Educ. 2017 Dec 4; 17 (1): 239. doi: 10.1186/s12909-017-1037-6 29202736 PMC5715628

[17] Marton GE, McCullough B, Ramnanan CJ. A review of teaching skills development programmes for medical students. BMC Medical Educ. 2015; 49(2):149–60. doi: 10.1111/medu.12571 25626746

[18] Alvarez S, Schultz JH. Practice Report/Bericht aus der Praxis: An exploration of peer tutor roles and recruitment at German medical schools. ZEFQ. 2017 Nov 127–128: 80–84. doi: 10.1016/j.zefq.2017.10.004 29128429

[19] Heni M, Lammerding-Köppel M, Celebi N, Shiozawa T, Riessen R, Nikendei C, et al. Focused didactic training for skills lab student tutors–which techniques are considered helpful? GMS J Med Educ. 2012; 29(3). Doc41. doi: 10.3205/zma000811 22737196 PMC3374137

[20] Reichel K, Dietsche S, Hölzer H, Ewers M. Interprofessional peer-assisted learning as a low-threshold course for joint learning: Evaluation results of the interTUT Project. GMS J Med Educ. 2016; 33(2), Doc30. doi: 10.3205/zma001029 27280141 PMC4895850

[21] Alvarez S, Dethleffsen K, Esper T, Horneffer A, Reschke K, Schultz J-H. An overview of peer tutor training strategies at German medical schools. ZEFQ. 2017 Oct 126: 77–83. doi: 10.1016/j.zefq.2017.09.009 29132600

[22] Herinek D, Woodward-Kron R, Ewers M: Preparing Tutors for Interprofessional Peer-Assisted Learning in Health Professions Education (Prep4TUT)–A Mixed-Methods Study Protocol. J. Interprof. Care. Forthcoming 2022. doi: 10.1080/13561820.2022.2066072 35543320

[23] Persky AM, McLaughlin JE. The Flipped Classroom–From Theory to Practice in Health Professional Education. Am J Pharm Educ. 2017 Aug 81(6): 118. doi: 10.5688/ajpe816118 28970619 PMC5607728

[24] Chacko TV. Blended learning in the 21st century: The need to tailor it to the changing learner self-direction levels during different phases of health professions education and beyond. Arch Med Health Sci. 2021; 9: 16–18. doi: 10.4103/amhs.amhs_117_21

[25] Ewers M, Paradis E, Herinek D. Interprofessionelles Lernen, Lehren und Arbeiten. Gesundheits- und Sozialprofessionen auf dem Weg zu kooperativer Praxis. Weinheim: Beltz Juventa; 2018.

[26] Abu-Rish E, Kim S, Choe L, Varpio L, Malik E, White AA, et al. Current trends in interprofessional education of health sciences students: A literature review. J. Interprof. Care. 2012 Nov 26(6): 444–451. doi: 10.3109/13561820.2012.715604 22924872 PMC7594101

[27] Homeyer S, Hoffmann W, Hingst P, Oppermann RF, Dreier-Wolfgramm A. Effects of interprofessional education for medical and nursing students: enablers, barriers and expectations for optimizing future interprofessional collaboration–a qualitative study. BMC Nurs. 2018 Apr 10; 17:13. doi: 10.1186/s12912-018-0279-x 29643742 PMC5891914

