# Supplementary figures and images for: Interprofessional peer-assisted learning and tutor training practices in health professions education–A snapshot of Germany
Source: PLoS One. 2022 Dec 14;17(12):e0278872. doi: 10.1371/journal.pone.0278872 (PMC9749977; doi:10.1371/journal.pone.0278872)

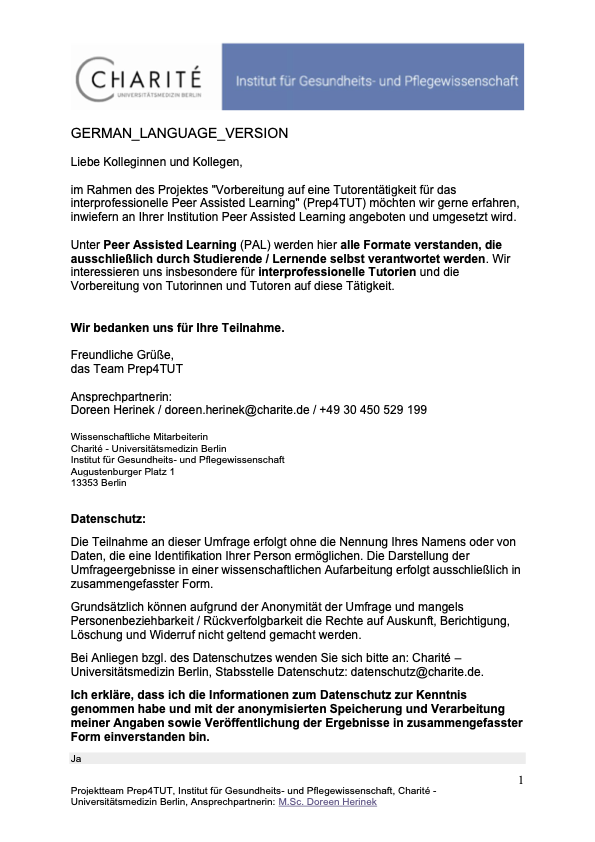

Supplement: S1 File — (TIFF) [file pone.0278872.s001.tiff]
